# Rat pancreatectomy combined with isoprenaline or uninephrectomy as models of diabetic cardiomyopathy or nephropathy

**DOI:** 10.1038/s41598-020-73046-8

**Published:** 2020-09-30

**Authors:** Louise Thisted, Mette V. Østergaard, Annemarie A. Pedersen, Philip J. Pedersen, Ross T. Lindsay, Andrew J. Murray, Lisbeth N. Fink, Tanja X. Pedersen, Thomas Secher, Thea T. Johansen, Sebastian T. Thrane, Torben Skarsfeldt, Jacob Jelsing, Morten B. Thomsen, Nora E. Zois

**Affiliations:** 1 In Vivo Pharmacology, Gubra Aps, Kongevej 11b, 2970 Hørsholm, Denmark; 2 Department of Biomedical Sciences, University of Copenhagen, Copenhagen, Denmark; 3 Department of Physiology, Development and Neuroscience, University of Cambridge, Cambridge, UK; 4 Serodus ASA, Oslo, Norway; 5 Present Address: CVRM, AstraZeneca, Gaithersburg, MD USA; 6 Present Address: CVD Research, Novo Nordisk, Måløv, Denmark; 7 Present Address: Department of Biomedicine, Aarhus University, Aarhus, Denmark

**Keywords:** Diabetes complications, Diabetes

## Abstract

Cardiovascular and renal complications are the predominant causes of morbidity and mortality amongst patients with diabetes. Development of novel treatments have been hampered by the lack of available animal models recapitulating the human disease. We hypothesized that experimental diabetes in rats combined with a cardiac or renal stressor, would mimic diabetic cardiomyopathy and nephropathy, respectively. Diabetes was surgically induced in male Sprague Dawley rats by 90% pancreatectomy (Px). Isoprenaline (Iso, 1 mg/kg, sc., 10 days) was administered 5 weeks after Px with the aim of inducing cardiomyopathy, and cardiac function and remodeling was assessed by echocardiography 10 weeks after surgery. Left ventricular (LV) fibrosis was quantified by Picro Sirius Red and gene expression analysis. Nephropathy was induced by Px combined with uninephrectomy (Px-UNx). Kidney function was assessed by measurement of glomerular filtration rate (GFR) and urine albumin excretion, and kidney injury was evaluated by histopathology and gene expression analysis. Px resulted in stable hyperglycemia, hypoinsulinemia, decreased C-peptide, and increased glycated hemoglobin (HbA1c) compared with sham-operated controls. Moreover, Px increased heart and LV weights and dimensions and caused a shift from α-myosin heavy chain (MHC) to β-MHC gene expression. Isoprenaline treatment, but not Px, decreased ejection fraction and induced LV fibrosis. There was no apparent interaction between Px and Iso treatment. The superimposition of Px and UNx increased GFR, indicating hyperfiltration. Compared with sham-operated controls, Px-UNx induced albuminuria and increased urine markers of kidney injury, including neutrophil gelatinase-associated lipocalin (NGAL) and podocalyxin, concomitant with upregulated renal gene expression of NGAL and kidney injury molecule 1 (KIM-1). Whereas Px and isoprenaline separately produced clinical endpoints related to diabetic cardiomyopathy, the combination of the two did not accentuate disease development. Conversely, Px in combination with UNx resulted in several clinical hallmarks of diabetic nephropathy indicative of early disease development.

## Introduction

Diabetes is a global disorder presently affecting 463 million people worldwide and the prevalence is increasing at alarming rates^1^. Moreover, diabetes is strongly associated with both cardiovascular and renal complications, which are significantly implicated in premature death among these patients^2,3^. Long-term diabetes increases the risk of cardiovascular disease, including myocardial infarction, stroke, and hypertension^4–6^. Independently, patients with diabetes also experience structural and functional abnormalities of the myocardium defined as diabetic cardiomyopathy (DbCM)^7,8^. The clinical manifestation of DbCM includes myocardial dilatation and hypertrophy, left ventricular (LV) dysfunction, and interstitial fibrosis^8–10^. The underlying mechanism is still incompletely understood but aside from metabolic derangements, microvascular dysfunction has been implicated^7,11^. Microvascular changes are also implicated in diabetic nephropathy (DN), which occurs in up to 40% of patients with diabetes. In addition to being associated with increased cardiovascular mortality, DN is also the leading cause of chronic and end-stage kidney disease^12^. The main clinical manifestations of DN are persistent albuminuria and decreased glomerular filtration rate (GFR)^13^, while additional hallmarks include renal hypertrophy, loss of podocytes, glomerulosclerosis, and tubulointerstitial fibrosis^14,15^. Nonetheless, DN is a heterogeneous kidney disease exhibiting variability in the degree of albuminuria, histopathological features, and different disease trajectories.

Further insight into the pathogenesis of both DbCM and DN is essential in order to advance clinical management of these major causes of morbidity and mortality worldwide. According to the Diabetic Complications Consortium, a valid rodent model of DbCM should include LV dysfunction and hypertrophy, interstitial fibrosis, and altered gene expression, but also an increased vulnerability to cardiac stressors^16^. For DN, a valid model should comprise progressive albuminuria and GFR loss, alongside characteristic histopathological changes such as arteriolar hyalinosis, glomerulosclerosis, and tubulointerstitial fibrosis^17^. The most-studied, current rodent models of diabetic complications are often limited in their usefulness as they produce only a few essential features associated with early stages of DbCM and DN progression^18–20^. Some of these commonly-used models of diabetes, e.g. streptozotocin-treatment (STZ) and the *db/db* genetic mouse model, have previously been combined with angiotensin II or uninephrectomy to mimic and accelerate DbCM or DN, respectively^21–23^.

The use of STZ in this context is however suboptimal, since the toxin also has the potential to induce direct nephrotoxic and cardiotoxic effects, independent of hyperglycemia^24–26^. Pancreatectomy (Px), on the other hand, is a well-characterized method of surgically inducing diabetes in rats^27^. An important advantage of this model is that it reflects the isolated effects of a reduced beta cell mass; immediately after surgery Px rats become insulin deficient and hyperglycemic, allowing full control of diabetic onset^28^. To our knowledge, the cardiac and renal pathology has not previously been evaluated in the Px rat nor have models of DbCM and DN been superimposed upon this model. The aim of this study was therefore to investigate the development and progression of DbCM and DN in a Px rat model of diabetes. To accentuate the cardiac and renal changes, we combined Px with isoprenaline, a non-selective β-adrenoreceptor agonist and well-known cardiac stressor capable of inducing cardiac dysfunction and fibrosis^29–31^, and uninephrectomy, which is known to accelerate the progression of injury in the remaining kidney^32,33^. We hypothesized that superimposing these cardiac or renal stressors on pre-existing diabetes would result in rat models that recapitulated key features of human DbCM and DN, respectively.

## Materials and methods

### Animals

Male Sprague–Dawley rats (NTac:SD) (8–10 weeks old, 210–280 g, Taconic Biosciences) were single-housed in a controlled environment (20–22 °C, humidity 40–60%) with a 12 h light/dark cycle. The animals were acclimatized for at least 1 week before surgery. All animals had ad libitum access to regular chow diet (Altromin 1324, Brogaarden) and tap water throughout the study period. The studies were approved by The Danish Animal Experiments Inspectorate (license no. 2019-15-0201-01648, 2017-15-0201-01183, 2017-15-0201-01286) and conformed to the European Parliament Directive on the Protection of Animals Used for Scientific Purposes (2010/63/EU).

### Experimental design

**Figure 1:**
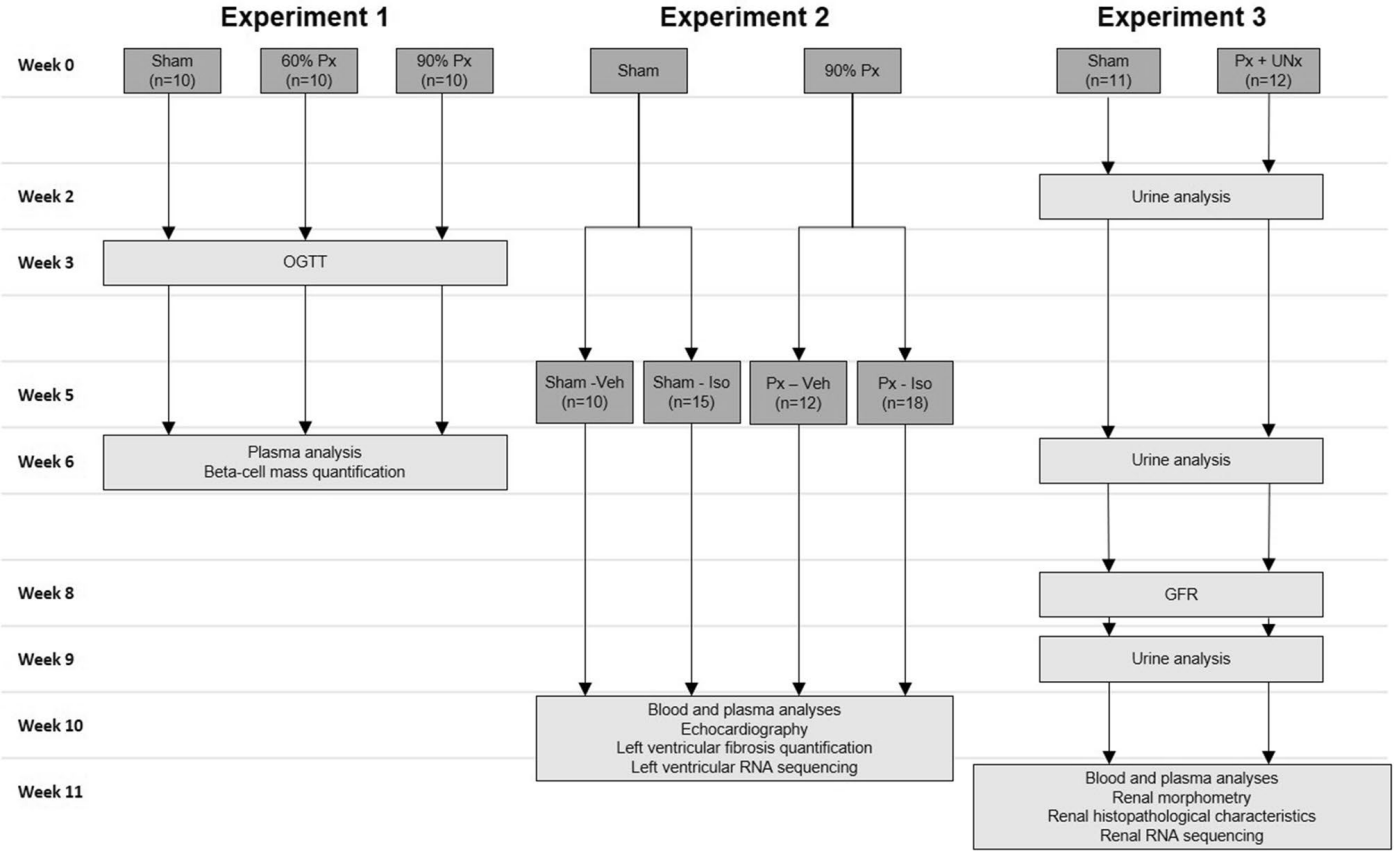
Study design of experiment 1–3. GFR: glomerular filtration rate, Iso: isoprenaline, OGTT: oral glucose tolerance test, Px: pancreatectomy, Veh: vehicle, UNx: uninephrectomy.

Three separate experiments were conducted in three cohorts of rats (Fig. 1). Experiment 1 involved metabolic characterization of the Px rat model and included sham surgery (n = 10), 60% Px (n = 10), and 90% Px (n = 10). Experiment 2 evaluated the 90% Px and isoprenaline-treated rat as a model of DbCM, and included sham-vehicle (Sham-Veh, n = 10), Px-vehicle (Px-Veh, n = 15), sham-isoprenaline (Sham-Iso, n = 12), and Px-isoprenaline (Px-Iso, n = 18) groups. Experiment 3 investigated the renal phenotype of the 90% Px and uninephrectomized rat model of DN in sham-operated (n = 11) and Px and uninephrectomized rats (Px-UNx, n = 12). In experiments 2 and 3, only Px rats exhibiting fasted or fed blood glucose above 10 or 12 mmol/L, respectively, 2 weeks post-Px were included.

### Pancreatectomy

Rats were fasted overnight and received subcutaneous injections of atropine (0.1 mg/kg), enrofloxacin (50 mg/kg), carprofen (50 mg/kg) and saline (20 ml/kg) prior to surgery. All surgical interventions were performed under isoflurane anesthesia (2–3%). The rats were fasted until the next day, whereupon 5 g of chow was administered to each animal. Two days after surgery, the animals were fed ad libitum.

#### Pancreatectomy

Through a midline abdominal incision, the pancreatic tissue was removed by gentle abrasion with disposable micro-brushes (Plandent). All major blood vessels were left intact. For 60% Px, the tail and body of the pancreas were removed whereas the pancreatic tissue within the duodenal loop, comprising the entire head of the pancreatic tissue, was preserved. For 90% Px, only the pancreatic tissue between the common bile duct extending to the first loop of the duodenum was preserved.

#### Sham surgery

Through a midline abdominal incision, the tail and body of the pancreatic tissue was disengaged from the mesentery and gently manipulated before being repositioned to the abdominal cavity.

### Isoprenaline treatment

Isoprenaline hydrochloride (Sigma-Aldrich) was dissolved in saline immediately prior to dosing and administered subcutaneously (1 mg/kg) for ten consecutive days beginning 5 weeks after sham surgery or 90% Px.

### Uninephrectomy

Animals were subjected to uninephrectomy at the time of 90% Px. The right ureter, renal artery and vein were identified and ligated. Subsequently, the right kidney was removed. In sham-operated animals, the right kidney was exposed and gently manipulated.

### Blood and plasma analyses

#### Blood glucose

Tail vein blood was collected once weekly from non-fasted or fasted (4 h) rats into heparinized glass capillary tubes and immediately suspended in glucose/lactate system solution buffer (EKF-diagnostics). The glucose concentration was measured immediately using a BIOSEN c-Line glucometer (EKF-diagnostics) according to the manufacturer’s instructions.

#### Oral glucose tolerance test (OGTT)

In experiment 1, animals were fasted for 4 h prior to oral glucose administration (2 g/kg body weight, 500 mg glucose/mL, Fresenius Kabi), and blood glucose was measured at 6 timepoints over the course of 240 min post-injection.

#### Blood and plasma markers

Plasma insulin and C-peptide were measured in duplicate using AlphaLISA platform (Perkin Elmer) according to the manufacturer’s instructions. Glycated haemoglobin (HbA1c), plasma creatinine, and urea were measured using commercial assays (Roche Diagnostics) on the Cobas C-501 autoanalyzer, and plasma cystatin C was measured using a commercial assay (R&D Systems) as per manufacturers’ instructions.

### Urine collection and biochemical analyses

Rats were single-housed in metabolic cages (Techniplast) with free access to powdered chow and water. The excreted urine was collected for 16 h. Urine creatinine was quantified using a CREP2 assay (Roche Diagnostics) on the Cobas C-501 autoanalyzer as per manufacturer’s instructions. Urine albumin was measured using a rat albumin ELISA (Bethyl Laboratories). Urine neutrophil gelatinase-associated lipocalin (NGAL), soluble tumor necrosis factor receptors (sTNFR) I and II were measured using ELISA assays (R&D Systems), while urine nephrin and podocalyxin were quantified using ELISA assays from LSBio and CusaBio, respectively. All ELISA assays were run as per manufacturers’ instructions and levels of analytes were reported as the analyte-to-creatinine ratio (ACR) in urine. Urine ACR values were log10-transformed before statistical analyses.

### Echocardiography

Echocardiographic assessments were performed under isoflurane anesthesia (2–3%). After chest hair removal, the rats were positioned in supine position on a heated pad. Electrocardiogram electrodes (lead II configuration, 3M) were placed and the rectal temperature was measured before and after examination. All examinations were performed using an ultrasound device (Philips, iE33) with a 12 MHz sector array probe. Function and dimensions of the LV were assessed from parasternal short and apical long axis views. The LV internal diameter (LVID), anterior and posterior wall thickness (LVAW, LVPW) were assessed in systole (LVIDs, LVAWs and LVPWs, respectively) and diastole (LVIDd, LVAWd and LVPWd, respectively) using 2D-guided M-mode in a short axis view at the level of the papillary muscles, according to the leading edge to leading edge principle^34^. The ejection fraction (EF) was calculated using the Teichholz formula^35^. To correct for the difference in LV size, all short axis measurements were normalized to body weight. LV filling (E and A velocity and deceleration time) was measured in the apical 4-chamber long axis view with the sample volume placed at the tip of the mitral valve leaflets using pulsed wave Doppler. Echocardiographic analyses were performed offline using Q-station software (version 3.8.5, Philips). Image acquisitions and analyses were performed by one operator blinded towards identity of the animals.

### Glomerular filtration rate measurement

GFR was assessed by a fluorescein isothiocyanate (FITC)-inulin test. Briefly, a 5% solution of FITC-inulin (TdB Consultancy) was prepared in 0.85% saline by heating and overnight dialysis using a Spectra/Por 6 dialysis tube (1 kDa molecular cut-off; Spectrum Labs). The FITC-inulin solution was sterile filtered (0.2 µm syringe filter) before tail vein injections in conscious rats (100 mg/kg). Sublingual blood was collected in heparinized tubes at 8 timepoints over the course of 75 min post-injection. Plasma FITC-inulin concentrations were measured using a plate-reader (CLARIOstar, BMG LABTECH) and GFR was calculated using a two-compartment model from the rate of decay in plasma FITC-inulin as described previously^36^ and normalized to body weight.

### Histology and stereology

#### Pancreas

At termination, pancreatic samples from sham, 60% and 90% Px rats were removed *en-bloc* and incubated in 10% neutral buffered formalin until further processing. Pancreatic tissue was carefully dissected, weighed, and processed as previously described^37^. Briefly, pancreas was rolled tightly into strips of gauze before infiltration with paraffin in an automated tissue processor (VIP5, Sakura). The pancreas was then cut into 7–9 systematic uniform random tissue slabs with a razor blade fractionator and embedded in paraffin blocks with the cut surface down. Subsequently, 5 µm thick sections were cut from each block on a microtome and collected on microscope slides. Immunohistochemistry against beta cells and non-beta cells were performed as a double staining using standard procedures. Briefly, after deparaffinization and microwave oven pretreatment in Tris-EGTA buffer (pH 9), sections were stained for non-beta cells using an antibody cocktail consisting of rabbit anti-glucagon (Phoenix, H-028–02, 1:5000), rabbit anti-somatostatin (Dako, 0566, 1:7500), and rabbit anti-pancreatic polypeptide (Europroxima, B32-1, 1:5000). The antibody cocktail was detected using Envision + anti-rabbit HRP-coupled polymer system (Dako, K4002) and developed with DAB-Nickel as chromogen. Next, beta cells were stained using a guinea pig anti-insulin antibody (Dako, A0564, 1:6000) followed by a biotinylated secondary donkey anti-guinea pig antibody (Jackson ImmunoResearch 706-065-148, 1:2000). Amplification and development of the insulin staining was performed using the Vectastain ABC elite kit (Vector Laboratories PK6100) and Impact NovaRed (Vector Laboratories, SK-4805). Finally, sections were counterstained with Mayer’s hematoxylin (Sigma-Aldrich).

#### Heart and kidney

One half of the sagittally-divided LV or left kidney were fixed in 10% neutral buffered formalin for 24 h at room temperature. The fixed tissues were cut into 5–7 systematic uniform random tissue slabs with a razor blade fractionator and embedded in paraffin blocks with the cut surface down. For Picro Sirius Red staining, a set of 3 µm sections were cut on a microtome from each block and collected on microscope slides. For Periodic acid-Schiff (PAS) staining, another set of sections were cut in pairs with a dissector distance of 30 µm. Picro Sirius Red staining: After deparaffinization, sections were incubated in Wiegert’s iron hematoxylin (Sigma-Aldrich) and then stained in Picro Sirius Red (Sigma-Aldrich) before they were cover slipped with Pertex. PAS staining: sections were deparaffinized and oxidized with 0.5% periodic acid solution followed by incubation with Schiff's reagent. Sections were counterstained in Mayer's hematoxylin and cover slipped with Pertex. Podocin/type IV collagen double fluorescent immunohistochemical staining of kidney sections was performed as previously described^22^.

#### Image analysis and stereological quantification

Stereological quantification of beta cell and non-beta cell mass was calculated as an area fraction (area of beta cells versus total pancreatic area) multiplied by the dissected pancreas mass as previously described^37^. Heart and kidney collagen content was determined as Picro Sirius Red area fraction using image analysis. Stained slides were scanned under a 20 × objective in an Aperio Scanscope AT slide scanner and imported into an image analysis module in Visiopharm Integrator System (Visiopharm). A Bayesian classifier was trained to detect Picro Sirius Red positive collagen versus other tissue components. The collagen area fraction was calculated as the Picro Sirius Red area divided by the total tissue area. Intra-glomerular collagen type 4 content and stereological estimation of kidney compartmental volumes were determined as previously described^22^. The mean glomerular volume was calculated as total glomerular volume divided by the mean number of glomeruli estimated using the physical dissector on serially cut reference and look-up sections with a dissector distance of 30 µm^38^.

### RNA sequencing

Transcriptome analysis was performed by sequencing of RNA extracts from LV tissue and renal cortex. RNA was purified from homogenized tissue using the NucleoSpin RNA Plus kit (Macherey-Nagel GmbH). The RNA quantity was measured using Qubit (Thermo Scientific, Eugene, OR) and RNA quality was determined using a bioanalyzer with RNA 6000 Nano kit (Agilent). Purified RNA from each sample was used to generate a single end cDNA library using the NEBNext Ultra II Directional RNA Library Prep Kit for Illumina (New England Biolabs, Ipswich, MA). cDNA libraries were sequenced on a NextSeq 500 using NextSeq 500/550 High Output Kit V2 (Illumina) for 75 cycles to a depth of 14–28 million reads per sample. Reads were aligned to the Ensembl Rnor_6.0 release 89 *Rattus norvegicus* genome using STAR v.2.5.2a with default parameters^39^.

### Statistical analysis

Except for the gene expression data set, which was analysed using DEseq2 package for R with default parameters^40^, all data were analyzed using GraphPad Prism software (version 8.2). Normal distribution of data was assessed by inspection of QQ plots and by using the Shapiro–Wilk test. Normally distributed data are shown as mean ± standard error of mean (SEM). In experiment 1, repeated measures two-way analyses of variance (ANOVA) with Bonferroni’s post-hoc test were used for time-course data and one-way ANOVA with Tukey’s post-hoc test was used for insulin and beta cell mass data. In experiment 2, two-way ANOVA was applied and in addition to evaluating the main effects of Px and isoprenaline treatment, the interaction between the two was included to evaluate a potential synergistic effect. Main effects of Px or isoprenaline were reported when no interaction between these two was found. Unpaired *t*-test and repeated measurements two-way ANOVA with Bonferroni’s Multiple Comparisons test were used in experiment 3. A *p* value < 0.05 was considered statistically significant.

### Ethics approval and consent to participate

The animal experiments were approved by The Danish Animal Experiments Inspectorate (license no. 2019-15-0201-01648, 2017-15-0201-01183, 2017-15-0201-01286) and conformed to the European Parliament Directive on the Protection of Animals Used for Scientific Purposes (2010/63/EU).

### Consent for publication

All authors have declared their consent for this publication.

## Results

### Metabolic characterization of the pancreatectomized rat

**Figure 2:**
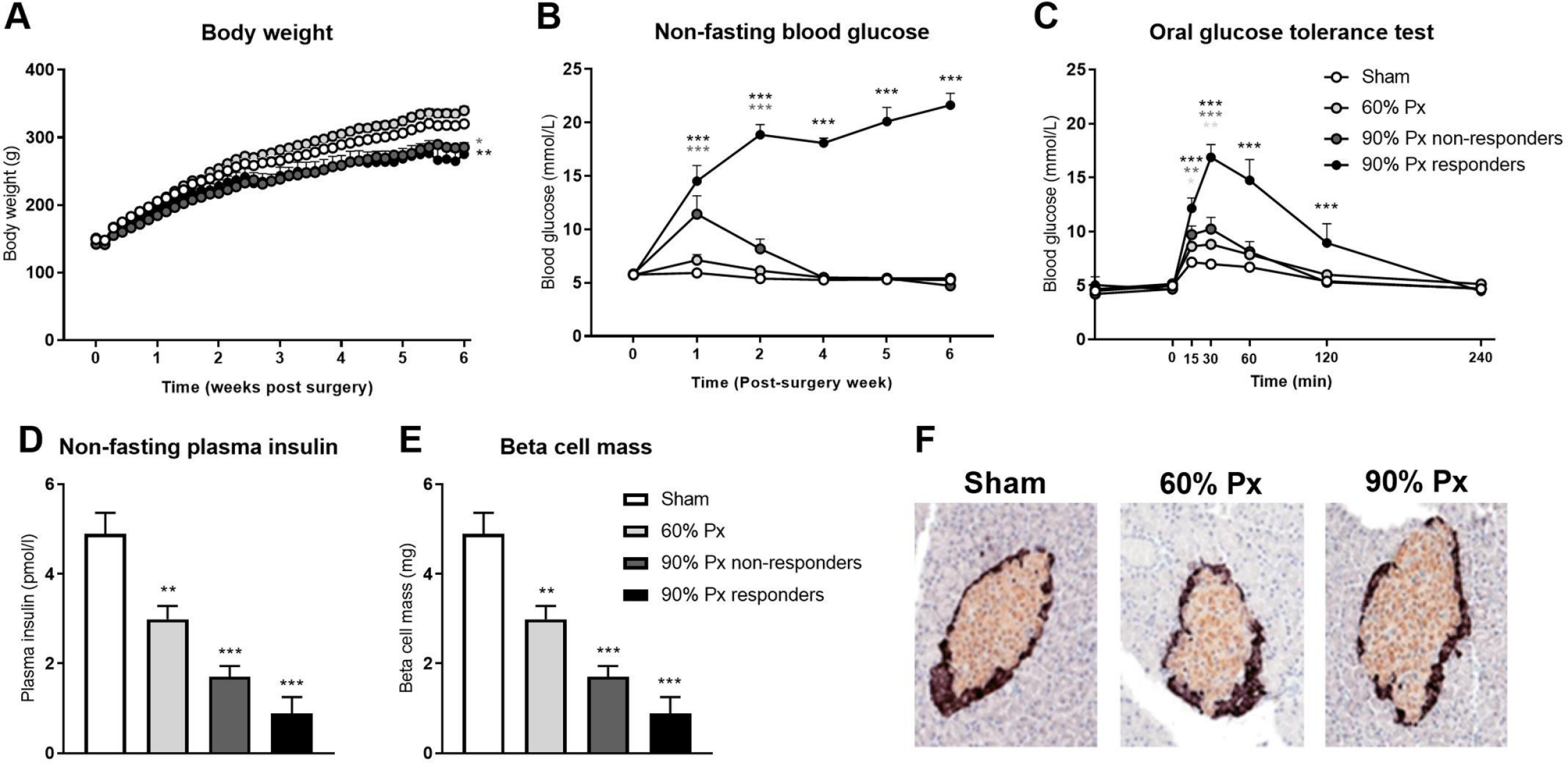
Body weight, blood glucose, plasma insulin, and beta cell mass for pancreatectomized animals. Body weight (**A**) and non-fasting blood glucose (**B**) measured in rats subjected to pancreatectomy (Px), where either 60% Px or 90% Px was removed, or sham surgery throughout the study period. Blood glucose levels from an oral glucose tolerance test performed 3 weeks after surgery (**C**). Non-fasting plasma insulin and total beta-cell mass determined by stereology 6 weeks after surgery (**D**, **E**) and representative images of sections from sham and Px rats stained for beta cells (orange-brown; insulin-immunoreactive) and non-beta cells (black; pancreatic polypeptide, somatostatin and glucagon-immunoreactive) (**F**). Data is presented as mean + SEM. n = 10, 10, 5, 5. Two-way ANOVA with Bonferroni’s post-hoc test was applied to blood glucose data and one-way ANOVA with Tukey’s post-hoc test to insulin and beta cell mass data. *p < 0.05; **p < 0.01; ***p < 0.001 vs Sham. Graphs are generated using GraphPad Prism (v 8.2), https://www.graphpad.com.

In experiment 1, 60% Px did not affect body weight, whereas 90% Px resulted in a lower rate of body weight gain over 6 weeks compared with sham-operation (*p* < 0.05, Fig. 2A). One week after surgery, 90% Px rats exhibited hyperglycemia (non-fasting blood glucose > 10 mmol/L) whereas 60% Px and sham rats were normoglycemic (*p* < 0.05, Fig. 2B). Among the 90% Px rats, 50% remained hyperglycemic throughout the 6-week study period (defined as ‘90% Px responders’) whereas 50% returned to normoglycemia 3 weeks after surgery (defined as ‘90% Px non-responders’). Correspondingly, blood glucose was increased for 15–120 min after an oral glucose bolus during the OGTT in 90% Px responders compared to sham (*p* < 0.05, Fig. 2C), but only for 15–30 min in 90% Px non-responders and in 60% Px. Both 60% and 90% Px resulted in significantly lower plasma insulin compared with sham (*p* < 0.05, Fig. 2D). While the total beta cell mass in the remaining pancreatic tissue was reduced in 60% and 90% Px rats compared to sham-operated controls 6 weeks after surgery (*p* < 0.01 and *p* < 0.001, respectively, Fig. 2E), morphology of the individual islets looked similar across groups (Fig. 2F).

### The pancreatectomized and isoprenaline-treated rat as a model of DbCM

In experiment 2, one rat was excluded due to lack of hyperglycemia 2 weeks after Px, five rats died from post-operative complications, and five Sham-Iso rats died after the first administration of isoprenaline (isoprenaline-induced mortality rate: 29.4%).

**Figure 3:**
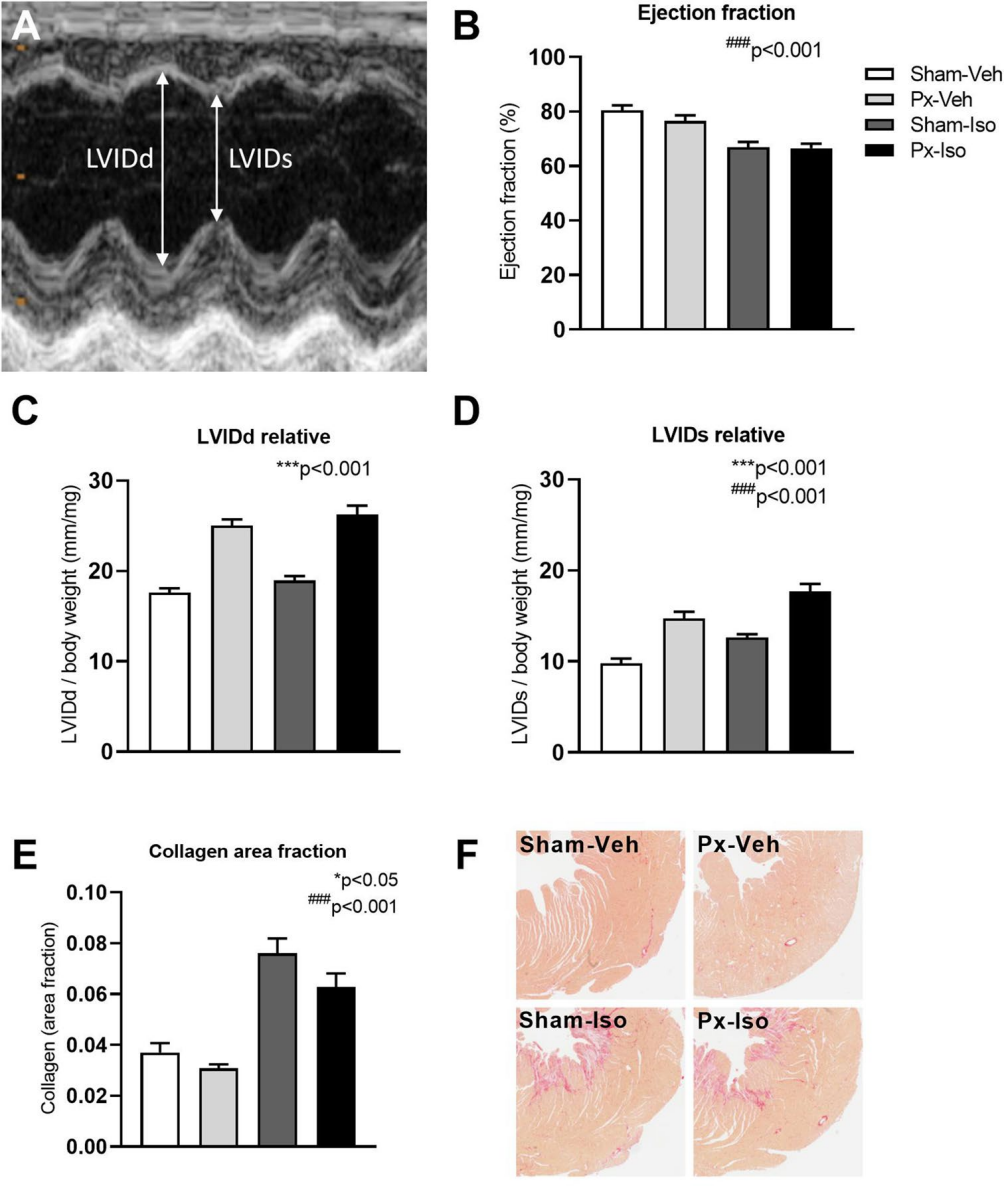
Echocardiographic and histological assessment of left ventricular (LV) morphology and function in pancreatectomized and isoprenaline treated rats. Echocardiographic parasternal short-axis views were used to obtain M-mode images at the level of the papillary muscles (**A**). LV ejection fraction (**B**) and end-diastolic and -systolic diameters (LVIDd, LVIDs) normalized to body weight (**C**, **D**), and collagen area fraction (**E**) were evaluated 10 weeks after sham surgery or pancreatectomy (Px) in vehicle (Veh)- or isoprenaline (Iso) treated rats. Representative images of Picro Sirius Red stained LV cross sections (**F**). Data is presented as mean + SEM. n = 10,15,12,18. Two-way ANOVA; main effect of pancreatectomy ***p < 0.001, main effect of isoprenaline treatment^###^*p* < 0.001. Graphs are generated using GraphPad Prism (v 8.2), https://www.graphpad.com.

No significant interaction between Px and isoprenaline treatment was found for any investigated outcome. Instead, irrespective of isoprenaline treatment, a main effect of Px was found for several measures reflecting glucose metabolism and LV remodeling. Specifically, HbA1c was increased post-Px, plasma C-peptide and insulin were decreased, and animals subjected to Px did not gain body weight to the same extent over the following 10 weeks as sham-operated animals (Table 1; all *p* < 0.001). Heart rate and peak E wave velocity were decreased (Table 2; *p* < 0.001) and relative heart and LV weight (*p* < 0.05), wall thickness and internal dimensions in systole and diastole (Fig. 3, Table 2; *p* < 0.001) were increased in Px rats compared with sham-operated counterparts. Furthermore, LV collagen area fraction was modestly decreased by Px (Fig. 3; *p* < 0.05). Owing to the frequent finding of fused E- and A-waves, calculation of E/A ratios was not possible during echocardiography.

**Table 1:** Baseline and terminal characteristics of pancreatectomized and isoprenaline treated rats. Measurements were performed at pre-surgery and/or 10 weeks after sham surgery or pancreatectomy (Px) in vehicle (Veh)- or isoprenaline (Iso) treated rats. Data is presented as mean ± SEM. Two-way ANOVA; main effect of pancreatectomy *p < 0.05; ***p < 0.001, main effect of isoprenaline treatment^###^*p* < 0.001.

	Sham-Veh (n = 10)	Px-Veh (n = 15)	Sham-Iso (n = 12)	Px-Iso (n = 18)	Main effect
Body weight (g, pre-surgery)	249 ± 5	253 ± 3	247 ± 6	248 ± 3	ns
Body weight (g, week 10)	454 ± 10	293 ± 11	447 ± 12	297 ± 10	***
Blood glucose (mmol/L, pre-surgery)	3.3 ± 0.2	3.6 ± 0.2	3.4 ± 0.1	3.4 ± 0.1	ns
Blood glucose (mmol/L, week 10)	4.8 ± 0.1	24.7 ± 1.0	4.7 ± 0.2	22.4 ± 1.5	***
Plasma insulin (pg/mL, week 10)	835 ± 96	180 ± 25	755 ± 87	173 ± 23	***
C-peptide (pg/mL, week 10)	2805 ± 105	839 ± 110	3012 ± 198	697 ± 70	***
HbA1c (%, week 10)	3.8 ± 0.1	9.2 ± 0.4	4.3 ± 0.5	10.0 ± 0.2	***
Heart weight (mg, week 10)	1459 ± 92	1259 ± 67	1206 ± 72	1244 ± 58	ns
Relative heart weight (mg/g body weight, week 10)	3.2 ± 0.2	4.2 ± 0.3	2.7 ± 0.1	4.2 ± 0.3	***
Left ventricular weight (mg, week 10)	966 ± 48	725 ± 30	1020 ± 36	760 ± 37	***
Relative left ventricular weight (mg/g body weight, week 10)	2.2 ± 0.1	2.4 ± 0.1	2.3 ± 0.1	2.5 ± 0.1	*
Kidney weight (mg, week 10)	1443 ± 50	1723 ± 77	1463 ± 79	1593 ± 88	*
Relative kidney weight (mg/g body weight, week 10)	3.1 ± 0.1	5.6 ± 0.2	3.4 ± 0.3	5.3 ± 0.2	***

**Table 2:** Echocardiographic assessment of left ventricular morphology and function in pancreatectomized and isoprenaline treated rats. Left ventricular wall thickness, filling parameters and heart rate ten weeks after sham surgery or pancreatectomy (Px) in vehicle (Veh)- or isoprenaline (Iso) treated rats. Heart rate was measured during echocardiography in week 10. Data is presented as mean ± SEM. Two-way ANOVA; main effect of pancreatectomy **p < 0.01; ***p < 0.001, main effect of isoprenaline treatment^#^*p* < 0.05.

	Sham-Veh (n = 10)	Px-Veh (n = 15)	Sham-Iso (n = 12)	Px-Iso (n = 18)	Main effect of Px or Iso
Relative LVAWd (mm/mg BW)	3.5 ± 0.3	5.1 ± 0.4	3.6 ± 0.2	4.8 ± 0.3	***
Relative LVPWd (mm/mg BW)	3.3 ± 0.3	4.4 ± 0.2	3.1 ± 0.2	4.2 ± 0.2	***
Relative LVAWs (mm/mg BW)	6.3 ± 0.3	8.9 ± 0.5	5.9 ± 0.3	7.6 ± 0.4	*** #
Relative LVPWs (mm/mg BW)	5.7 ± 0.4	6.9 ± 0.3	4.6 ± 0.3	6.3 ± 0.3	*** #
Peak E wave velocity (cm/s)	114 ± 3	107 ± 4	123 ± 5	104 ± 4	**
Deceleration time (ms)	42 ± 2	47 ± 5	41 ± 3	42 ± 3	ns
Heart rate (bpm)	368 ± 3	299 ± 8	373 ± 3	281 ± 18	***

In contrast to Px, isoprenaline treatment decreased indices of systolic LV function (LVIDs and EF) (Fig. 3, Table 2; both *p* < 0.001) and increased LV collagen area fraction (Fig. 3, *p* < 0.001).

**Figure 4:**
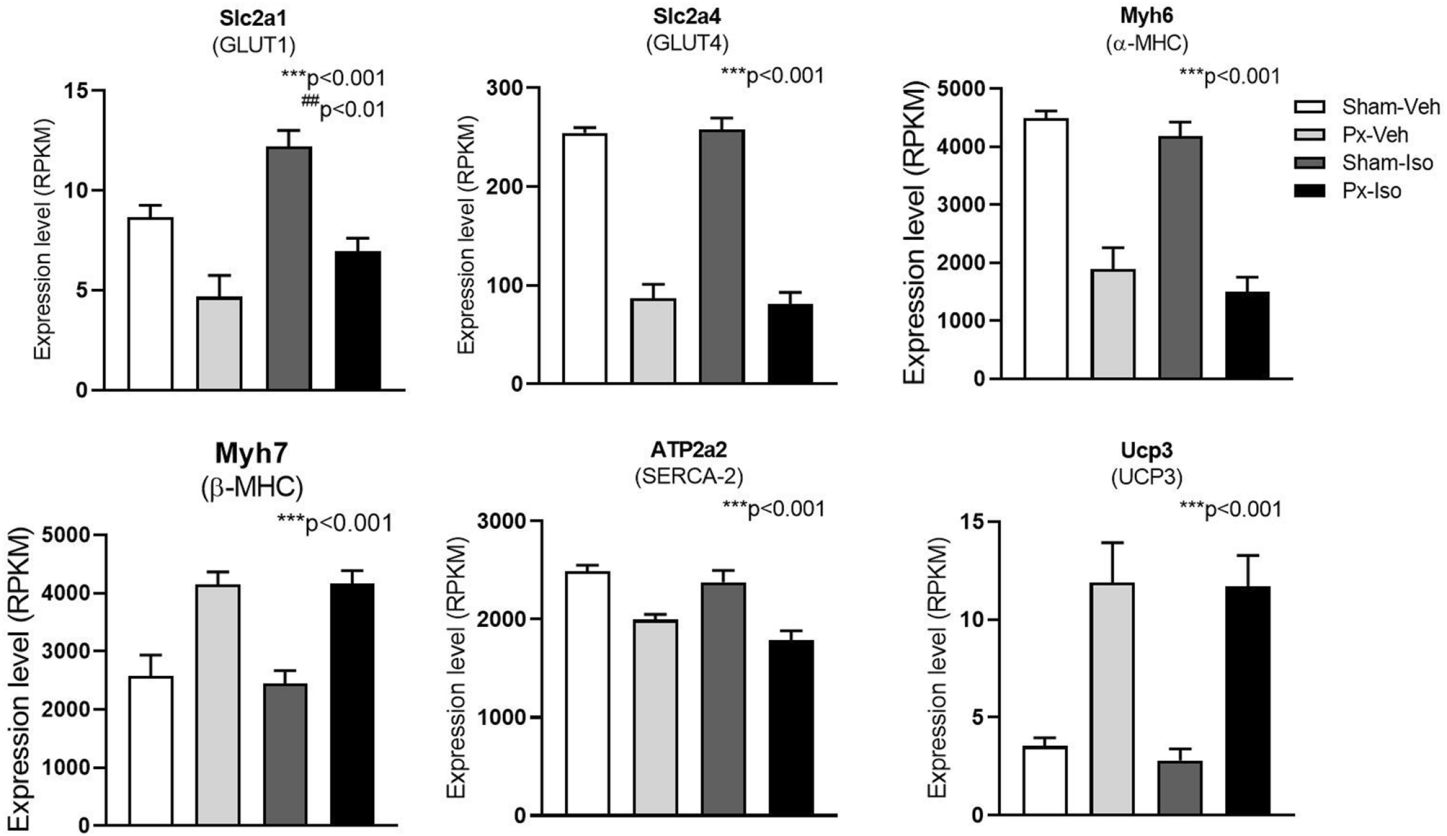
Expression of selected genes in the left ventricle determined by RNA sequencing of pancreatectomized and isoprenaline treated rats. Expression levels of genes relevant for cardiac function 10 weeks after sham surgery or 90% pancreatectomy (Px) in vehicle (Veh)- or isoprenaline (Iso) treated rats. Data is presented as mean + SEM. n = 7,8,8,8. Two-way ANOVA; main effect of pancreatectomy ***p < 0.001, main effect of isoprenaline treatment ^##^*p* < 0.01. GLUT: glucose transporter, MHC: myosin heavy chain. SERCA: sarco/endoplasmic reticulum Ca^2+^-ATPase, UCP: Uncoupling protein. Graphs are generated using GraphPad Prism (v 8.2), https://www.graphpad.com.

Transcriptome analysis of LV tissue revealed a Px-driven change in expression of genes related to cardiac metabolism and function compared to sham. Specifically, glucose transporter (GLUT) 1, GLUT4, α-myosin heavy chain (MHC), and sarco/endoplasmic reticulum Ca^2+-^ATPase (SERCA)-2 were all downregulated, whilst β-MHC and uncoupling protein (UCP) 3 were upregulated (*p* < 0.001, Fig. 4). In contrast, isoprenaline treatment per se did not affect expression of these selected genes compared to vehicle treatment other than upregulating GLUT1 (*p* < 0.01). Further analysis of expression levels of genes involved in adrenergic signaling revealed that β_2_-receptors were downregulated by Px (*p* < 0.01) while β_1_-receptors were unaffected (Supplementary Fig. S1). Moreover, expression of downstream mediators of β-receptor signaling, adenylate cyclase (AC) 6 and phosphodiesterase 3A (PDE3A) were upregulated by Px (*p* < 0.001 and *p* < 0.05, respectively) while PDE3A was downregulated by isoprenaline (*p* < 0.05, Supplementary Fig. S1).

### The pancreatectomized and uninephrectomized rat as a model of DN

In experiment 2, renal changes induced by Px were observed including increased left kidney weight (Table 1, *p* < 0.05) and urine albumin-to-creatinine ratio (2549 ± 1447 µg/mg vs. 64 ± 10 µg/mg, *p* < 0.01) in Px-Veh compared to Sham-Veh 10 weeks after surgery. This indicates presence of modest renal pathology in the Px rats and we therefore conducted a pilot study to investigate if UNx, a known accelerator of disease progression in rodent models of DN, exacerbated renal changes in Px rats. Here, we found that UNx increased urine markers of kidney injury (i.e. albumin and NGAL), kidney and glomerular hypertrophy, and glomerular collagen 4 in Px rats (see Supplementary Fig. S2). Subsequently in experiment 3, the progression of DN in Px rats was investigated in conjunction with UNx and compared to sham operated, age-matched rats. As expected, Px-UNx caused an increase in blood glucose and a decrease in plasma insulin compared to sham (both *p* < 0.05, Table 3). Furthermore, relative heart weight was increased in Px-UNx compared to sham (*p* < 0.001, Table 3). Urinary excretion of albumin, NGAL, and sTNFR-II was increased in Px-UNx 2 weeks after surgery and was sustained at 6 and 9 weeks post-surgery (all *p* < 0.001, Table 4). Urine sTNFR-I and podocalyxin excretion was increased by week six and nine after surgery (both *p* < 0.001). At termination, 11 weeks after surgery, plasma urea was increased in Px-UNx (*p* < 0.05), whereas plasma creatinine was reduced (*p* < 0.05). Cystatin C was not significantly different, but GFR was increased 8 weeks after surgery in Px-UNx compared to sham (*p* < 0.05).

**Table 3:** Terminal characteristics of pancreatectomized-uninephrectomized rats. Glomerular filtration rate was measured eight weeks after surgery. Body and heart weight, and blood and plasma levels at eleven weeks in sham operated or pancreatectomized-uninephrectomized (Px-UNx) rats. Data are presented as mean ± SEM. Unpaired *t* test; *p < 0.05; **p < 0.01; ***p < 0.001 vs. Sham.

	Sham (n = 12)	Px-UNx (n = 11)	
Glomerular filtration rate (ml/min/kg)	6.6 ± 0.3	8.2 ± 0.3	**
Body weight (g)	450 ± 3	306 ± 4	***
Blood glucose (mmol/L)	4.8 ± 0.1	26.2 ± 1.8	***
Insulin (pg/mL)	782 ± 102	193 ± 58	***
Urea (mmol/L)	5.5 ± 0.2	9.4 ± 0.7	***
Creatinine (µmol/L)	23.5 ± 0.6	21.2 ± 0.7	*
Cystatin C (ng/mL)	2373 ± 50	2370 ± 56	ns
Relative kidney weight (mg/g body weight)	3.2 ± 0.0	9.3 ± 0.1	***
Relative heart weight (mg/g body weight)	2.8 ± 0.0	3.3 ± 0.0	***

**Table 4:** Urine markers in pancreatectomized-uninephrectomized and sham rats. Measurements were performed after two, six, and nine weeks in sham operated or pancreatectomized-uninephrectomized (Px-UNx) rats. Data is presented as mean ± SEM. Two-way repeated measures ANOVA with Bonferroni’s Multiple Comparisons post-hoc test; **p < 0.01; ***p < 0.001 vs. Sham.

	Week 2			Week 6				Week 9			
	Sham (n = 12)	Px-UNx (n = 11)		Sham (n = 12)		Px-UNx (n = 11)		Sham (n = 12)		Px-UNx (n = 11)	
Creatinine (µmol/L)	7197 ± 661	993 ± 209	***	8189 ± 444		606 ± 85	***	8273 ± 445		520 ± 65	***
Albumin-to-creatinine (µg/mg)	109 ± 13	1250 ± 653	***	207 ± 60		10,281 ± 6239	***	370 ± 170		13,473 ± 7619	***
NGAL-to-creatinine (µg/mg)	3.27 ± 0.8	16.6 ± 2.2	***	3.84 ± 0.4		14.9 ± 1.7	***	2.69 ± 0.4		13.3 ± 1.9	***
Podocalyxin-to-creatinine (µg/mg)	19.9 ± 2.7	76.2 ± 9.3		14.6 ± 1.8		186.9 ± 59.3	***	11.8 ± 2.0		195.2 ± 41.9	***
sTNFR-I- to-creatinine (pg/mg)	36.0 ± 3.4	259 ± 32		23.6 ± 1.4		427 ± 117	***	21.3 ± 1.3		447 ± 133	***
sTNFR-II-to-creatinine (pg/mg)	2521 ± 197	5212 ± 413	***	1460 ± 122		3520 ± 391	***	1348 ± 163		2743 ± 414	**

**Figure 5:**
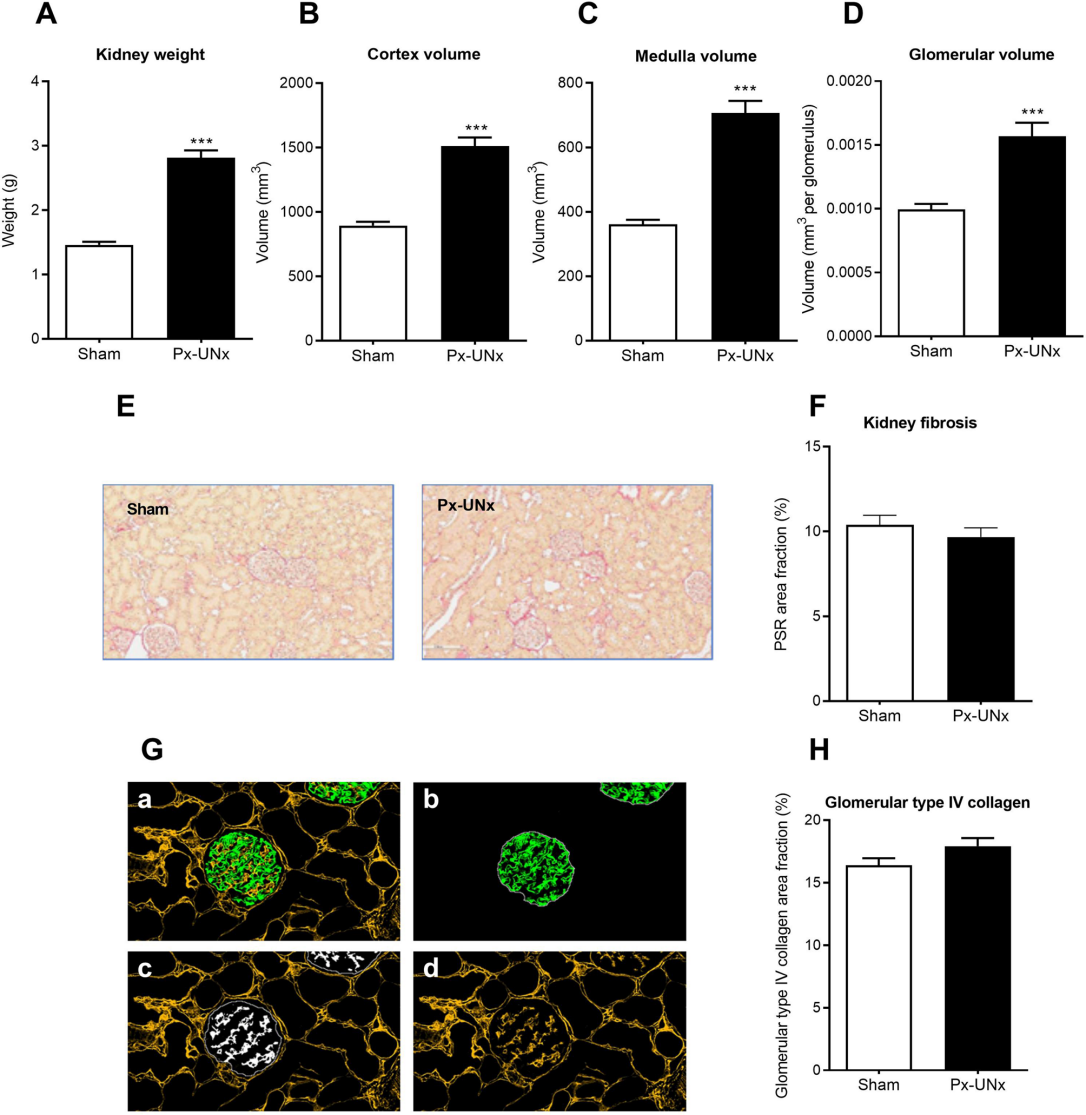
Renal morphometric and histopathological characteristics in pancreatectomized-uninephrectomized rats. Measurements were performed 11 weeks after sham or pancreatectomy-uninephrectomy (Px-UNx). Kidney weight and volume of renal compartments (A-D). Representative images of Picro Sirius Red (PSR) stained kidney sections and total renal fibrosis quantification (E–F). Representative images of kidney sections stained for co-localization type IV collagen (yellow) and podocin (green), and quantification of intra-glomerular type IV collagen (white) (G-H). Data is presented as mean + SEM. n = 12,11. Unpaired *t* test; ***p < 0.001 vs. sham. Graphs are generated using GraphPad Prism (v 8.2), https://www.graphpad.com.

Renal hypertrophy was observed in Px-UNx rats compared with sham-operated animals with increased kidney weight and renal cortex and medulla volumes (*p* < 0.05, Fig. 5A–C). Px-UNx rats also displayed significant glomerular hypertrophy as assessed by stereology (*p* < 0.05 vs. sham, Fig. 5D), in an unaltered number of glomeruli (39,472 ± 1336 vs 39,130 ± 1665 glomeruli, ns.). Fibrosis quantification by histopathology showed unchanged levels of renal collagen (Fig. 5E, F), and the area fraction of type IV collagen in the glomeruli was unaffected in Px-UNx rats compared with sham (Fig. 5G, H).

**Figure 6:**
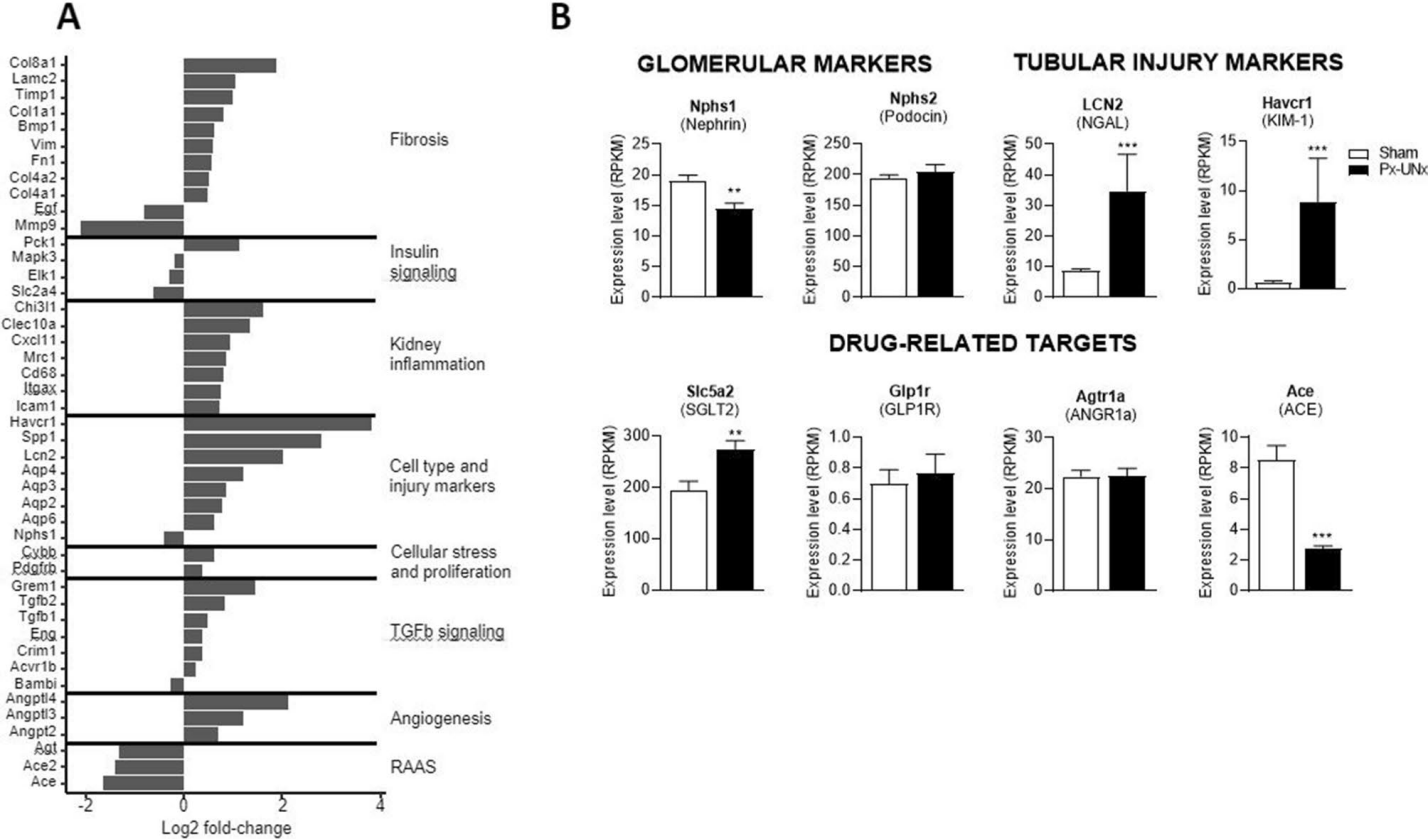
Renal gene expression determined by RNA sequencing in pancreatectomized-uninephrectomized rats. Measurements were performed at termination, 11 weeks after sham or pancreatectomy-uninephrectomy (Px-UNx) on RNA isolated from renal cortex. Pathway summary presenting selected genes that are significantly regulated in the renal cortex of Px-UNx vs. Sham animals at *p* < 0.01 significance level (A). Expression levels of selected genes in the renal cortex (B). Data are mean + SEM. n = 12,11. Unpaired *t* test; **p < 0.01; **p < 0.001 vs Sham after correction for gene-wise multiple testing. ACE: Angiotensin I converting enzyme, ANGR1a: Angiotensin II receptor, type 1a, GLP1R: Glucagon-like peptide 1 receptor, KIM-1: kidney injury molecule 1, NGAL: neutrophil gelatinase-associated lipocalin, RAAS: renin–angiotensin–aldosterone system, SGLT2: Sodium-glucose transport protein 2. Graphs are generated using ggplot2 in R (v 3.3.2), https://www.r-project.org and GraphPad Prism (v 8.2), https://www.graphpad.com.

Renal cortex gene expression analysis of selected pathways involved in the pathogenesis of DN is shown in Fig. 6A. Several genes involved in TGF-beta signaling, fibrosis and inflammation, and angiogenesis were upregulated, while the expression of genes involved in the renin–angiotensin–aldosterone system was downregulated (Fig. 6A). Genes of the insulin signaling pathway were significantly altered in Px-UNx compared to sham (Fig. 6A). Expression of podocyte markers was either down-regulated (nephrin) or unchanged (podocin), while tubular damage markers [neutrophil gelatinase-associated lipocalin (NGAL) and Kidney Injury Molecule-1 (KIM-1)] were significantly upregulated (*p* < 0.05, Fig. 6B). Finally, drug-related targets including sodium-glucose transport protein 2, glucagon-like peptide 1 receptor, and angiotensin I converting enzyme were differentially regulated in Px-UNx compared to sham (Fig. 6B).

## Discussion

Here we characterized two preclinical rat models of DbCM and DN by superimposing isoprenaline treatment and UNx, respectively, on Px. Px led to marked alterations in glucose homeostasis and induced morphological and functional changes related to cardiovascular and renal endpoints. While no synergistic effect was revealed by superimposing the cardiac stressor, isoprenaline, to Px, superimposing UNx to Px in rats led to exacerbated DN as measured by alterations in albuminuria and GFR.

In agreement with previous findings^41^, we demonstrated that 90%, but not 60%, Px induced permanent, stable hyperglycemia, concomitant with a surgically induced reduction in beta cell mass and thereby lower plasma insulin levels. Interestingly, we found that despite having undergone 90% Px, normoglycemia was restored 3 weeks after surgery in a subset (50%) of rats, termed non-responders, in experiment 1. These rats were able to maintain adequate insulin production to support normoglycemia. Hereafter, the Px procedure was further refined, which resulted in a reduction to 2% of non-responders in subsequent studies. Evidently, a certain threshold in critical beta cell mass exists rendering a meticulous use of anatomical landmarks during removal of pancreatic tissue a necessity.

Besides demonstrating a pronounced and sustained diabetic phenotype, including decreased C-peptide as well as increased HbA1c, the Px rat also exhibited mild cardiac hypertrophy and remodeling indicated by increased relative heart and LV weights, LV wall thickness and LVIDd. Although EF was preserved, early systolic dysfunction was revealed by increased LVIDs. In DbCM, diastolic dysfunction is known to precede systolic dysfunction but, in this study, unfortunately, several indices of diastolic function, including the peak velocity of the late filling of the LV, could not be derived due to low temporal resolution and thus E/A fusion during the echocardiographic examination. Although the LV filling pattern could not be fully evaluated, the finding of a decreased early filling, corresponding to the peak E wave mitral inflow velocity, may be indicative of impaired relaxation and thus early diastolic dysfunction in Px rats. Furthermore, Px intervention altered the expression of selected genes involved in the pathogenesis of DbCM as suggested by the Diabetes Complications Consortium^16^. For instance, the major cardiac glucose transporter, GLUT4, was downregulated in Px. Interestingly, impaired insulin signaling, including decreased recruitment of GLUT4 to the plasma membrane, has been implicated in promoting cardiomyocyte stiffness in the diabetic heart^42^. In support of an attenuated contractile function, a shift from the ‘fast’ isoform α-MHC to the slower β-MHC along with decreased SERCA-2 expression was found in LV tissue.

An important advantage of the Px model is that it reflects the isolated effects of reduced beta cell mass, without the potential multiple-organ toxic effects of STZ^24^ or the confounding effects of obesity and impaired leptin signaling found in e.g. ZDF rats. A disadvantage, however, is that not only beta cells, but also exocrine acinar cells are excised during 90% Px. The lack of pancreatic enzymes following Px^43^ may thus act as a confounder by potentially inducing malnutrition and growth retardation. In future studies, supplementation with pancreatic enzymes may therefore be considered to avoid such potential effects.

Isoprenaline is a β-adrenergic agonist promoting cardiac hypertrophy, fibrosis, and systolic dysfunction in rats when administered in supraphysiological dosages^31,44,45^. In the present study, isoprenaline treatment per se did not induce cardiac hypertrophy or remodeling but did induce increased LV fibrosis and reduced systolic function. A possible explanation for the lack of isoprenaline-induced LV hypertrophy is that the cardiac remodeling may be reversible as indicated previously by Golomb et al*.*^46^, demonstrating reversal just seven days after cessation of isoprenaline. In the present study, LV changes were assessed nearly 4 weeks after isoprenaline treatment and in comparison to previous studies where a single high-dose regimen has been used^30,44^, a relatively low isoprenaline dosage was applied.

Clinical studies of patients with diabetes have previously shown an increased susceptibility to hemodynamic stressors^47,48^ and we thus hypothesized that the cardiac changes induced by isoprenaline would be accelerated and accentuated when superimposed on Px. Unexpectedly, the combination of Px and isoprenaline treatment did not accentuate or accelerate LV morphological or functional changes but it cannot be excluded that molecular changes indicative of DbCM have been induced in this model.

Isoprenaline induced a nearly 30% mortality rate in sham-operated rats after the first exposure, whereas no isoprenaline-related deaths were observed in Px animals. Mortality following isoprenaline treatment has been reported previously using higher doses in non-diabetic rats^30,31,44^. In the present study, the expression of β_2_-receptors was slightly downregulated by Px whilst PDE3A was upregulated, which could attenuate responsiveness to β-receptor stimulation by isoprenaline^49^, and hence partly explain the lack of synergy, but also the increased mortality rate. Conversely, AC6, known to improve cardiac function and responsiveness to β-receptor stimulation^50^, was upregulated by Px. Another explanation could be related to the decreased heart rate in Px rats. Lower heart rates will have longer refractory periods and this phenomenon could perhaps protect against isoprenaline-induced tachycardia and arrhythmias. However, further experiments are needed to elucidate this subject further.

UNx is known to accelerate DN progression in rodents^18^, which we confirmed in our DN pilot study, and, importantly, we observed that the combination of Px and UNx induced several hallmarks of DN. Px-UNx rats displayed profound renal and glomerular hypertrophy, which together with increased GFR are indicative of renal hyperfiltration. These phenomena are also observed in patients during the early phase of DN disease progression^51^. Importantly, Px-UNx animals presented with persistent albuminuria detectable as early as 2 weeks post-surgery. Similarly, urine NGAL excretion was increased, which is indicative of tubular damage and supported by increased RNA expression levels of the clinical biomarkers, NGAL and KIM-1^52,53^. Urine sTNFR I and II excretion was increased; a finding that corresponds to reports of diabetic patients with diabetic kidney disease^54^. Finally, urine podocalyxin excretion was increased in line with observations of early podocyte injury in human patients^55^.

Although expression of genes involved in kidney fibrosis and inflammation (incl. extracellular matrix genes and TGF-beta signaling) was increased in the renal cortex of Px-UNx rats, histopathological evaluation of kidneys from the Px-UNx model did not reveal evidence of renal fibrosis or glomerulosclerosis as measured by intra-glomerular type IV collagen. On the contrary, stereological assessment of the kidney revealed massive renal and glomerular hypertrophy. Together with the measured GFR, these data demonstrate that the Px-UNx rat represents a model of early stage DN in human patients. Longer term studies, beyond the 10 weeks reported here, are likely required for progression of DN with resulting GFR loss, glomerulosclerosis, and tubulointerstitial fibrosis.

Renal gene expression in the Px-UNx rat showed significant induction of genes associated with fibrosis (incl. collagen 1a1, collagen 4a1, and Lamc2), TGF-beta signalling (incl. Grem1, TGF-beta 1 and 2), inflammation (incl. CD68 and ICAM1), and injury markers (incl. Havcr1 and Lcn2) reflecting the pro-fibrotic and pro-inflammatory milieu in the kidney of Px-UNx rats. Furthermore, expression of podocyte markers, Nphs1 and Nphs2, was reduced or not regulated in Px-UNx indicating that the loss of podocytes is limited in this model and supporting the notion that Px-UNx rats display features of early stage DN. Furthermore, the expression of drug-related target genes was also investigated and showed increased expression of SGLT2 in the diabetic kidney in line with findings in other rodent models of DN^56^. On the other hand, expression of GLP-1R was unaffected in Px-UNx. This is contradictory to findings in the STZ-induced diabetic mouse, where GLP-1R gene expression is found to be reduced in the glomerulus^57^. This discrepancy may be explained by the fact that the analysis of gene expression in experiment 3 was conducted by sequencing renal cortex RNA, and gene expression levels thus represent an average of the expression across glomerular, tubular, vascular and interstitial compartments. Seeing that glomeruli make up only approximately 1% of the total kidney volume in diabetic, uninephrectomized mice^58^, transcriptional changes of a gene in one compartment (e.g. the glomerulus) may thus be masked for genes expressed in more than one compartment such as e.g. GLP-1R. The ACE and ACE2 expression data underscores this notion; we show that cortical expression of ACE and ACE2 is decreased in Px-UNx, whereas compartmentalized gene expression analysis show increased expression of ACE and ACE2 in glomeruli and decreased expression in the tubulointerstitium and whole kidneys in STZ-induced diabetes in rats^59^.

Interestingly, in experiment 3, we found that the relative weight of the heart was increased almost 18% in Px-UNx rats compared to sham. Seeing that Px alone increased the weight of the left ventricle by only 9% this could be indicative of cardiorenal syndrome. However, cardiac changes in our DN model were beyond the scope of the present study, but a topic worth pursuing in a future study.

Although non-diabetic uninephrectomized animals were not included as a control in this study, our data suggest that UNx accelerates DN in the Px rat. Compared with Px rats from experiment 2, UNx-Px rats displayed augmented urine ACR in experiment 3. This suggests that kidney injury is accelerated in Px-UNx compared with Px alone.

## Conclusion

In conclusion, 90% Px in the rat resulted in a robust model of experimental diabetes with a surgically induced onset of hyperglycemia concomitant with hypoinsulinemia. The observation of increased LV wall thickness and internal diameters indicated that Px may mimic early stages of DbCM disease development. The preclinical applicability was supported by changes in expression levels in genes centrally involved in cardiomyocyte changes related to DbCM. However, the combination of isoprenaline and Px did not further accentuate DbCM development. Px in combination with UNx exhibited pronounced albuminuria, renal and glomerular hypertrophy together with increased GFR resembling features of early stage DN. The Px and the Px-UNx models could therefore be useful to study the potential efficacy of new therapeutic interventions for attenuating early disease progression in DbCM and DN, respectively.

## Supplementary information

Supplementary file 1.

## Data Availability

All data and materials are available upon request.

## Abbreviations

AC: Adenylate cyclase
ACR: Analyte-to-creatinine ratio
ANOVA: Analysis of variance
DbCM: Diabetic cardiomyopathy
DN: Diabetic nephropathy
EF: Ejection fraction
FITC: Fluorescein isothiocyanate
GFR: Glomerular filtration rate
GLUT: Glucose transporter
HbA1c: Glycated hemoglobin
Iso: Isoprenaline
KIM-1: Kidney Injury Molecule-1
LV: Left ventricular
LVAWd: Left ventricular anterior wall thickness in diastole
LVAWs: Left ventricular anterior wall thickness in systole
LVIDd: Left ventricular internal diameter in diastole
LVIDs: Left ventricular internal diameter in systole
LVPWd: Left ventricular posterior wall thickness in diastole
LVPWs: Left ventricular posterior wall thickness in systole
MHC: Myosin heavy chain
NGAL: Neutrophil gelatinase-associated lipocalin
OGTT: Oral glucose tolerance test
PAS: Periodic acid-Schiff
PDE3A: Phosphodiesterase 3A
Px: Pancreatectomy
SEM: Standard error of mean
SERCA: Sarco/endoplasmic reticulum Ca^2^^+^-ATPase
sTNFR: Soluble tumor necrosis factor receptor
STZ: Streptozotocin
UNx: Uninephrectomy
UCP: Uncoupling protein
