# Supplementary material for: Rat pancreatectomy combined with isoprenaline or uninephrectomy as models of diabetic cardiomyopathy or nephropathy
Source: Sci Rep. 2020 Sep 30;10:16130. doi: 10.1038/s41598-020-73046-8 (PMC7527487; doi:10.1038/s41598-020-73046-8)
Supplement: Supplementary file 1 [file 41598_2020_73046_MOESM1_ESM.docx]

**Supplementary material**

**Rat pancreatectomy combined with isoprenaline or uninephrectomy as models of diabetic cardiomyopathy or nephropathy**

Louise Thisted^1,2^, Mette V. Østergaard^1^, Annemarie A. Pedersen^1^, Philip J. Pedersen^1^, Ross T. Lindsay^1,3,*^, Andrew J. Murray^3^, Lisbeth N. Fink^1^, Tanja X. Pedersen^1,**^, Thomas Secher^1^, Thea T. Johansen^1,***^, Sebastian T. Thrane^1^, Torben Skarsfeldt^4^, Jacob Jelsing^1^, Morten B. Thomsen^2^, Nora E Zois^1^.

^1^Gubra Aps, Hørsholm, Denmark

^2^Dept. of Biomedical Sciences, University of Copenhagen, Copenhagen, Denmark

^3^Dept. of Physiology, Development and Neuroscience, University of Cambridge, Cambridge, UK.

^4^Serodus ASA, Oslo, Norway

*Current affiliation: CVRM, AstraZeneca, Gaithersburg, Maryland, USA

**Current affiliation: CVD Research, Novo Nordisk, Måløv, Denmark

***Current affiliation: Dept. of Biomedicine, Aarhus University, Aarhus, Denmark

Corresponding author: Nora Elisabeth Zois, Gubra Aps, In vivo pharmacology, Hørsholm Kongevej 11b, 2970 Hørsholm, Denmark, +45 6170 9037, nez@gubra.dk

**Supplementary figure 1. Expression of selected genes in the left ventricle determined by RNA sequencing of pancreatectomized and isoprenaline treated rats.** Expression levels of genes relevant for cardiac function ten weeks after sham surgery or 90% pancreatectomy (Px) in vehicle (Veh)- or isoprenaline (Iso) treated rats. Data is presented as mean + SEM. n=7,8,8,8. Two-way ANOVA across all groups; overall effect of pancreatectomy **p<0.01, ***p<0.001. ADCY6: adenylyl cyclase 6, ADRB: beta-adrenoceptor, PDE3A: phosphodiesterase 3a.

**
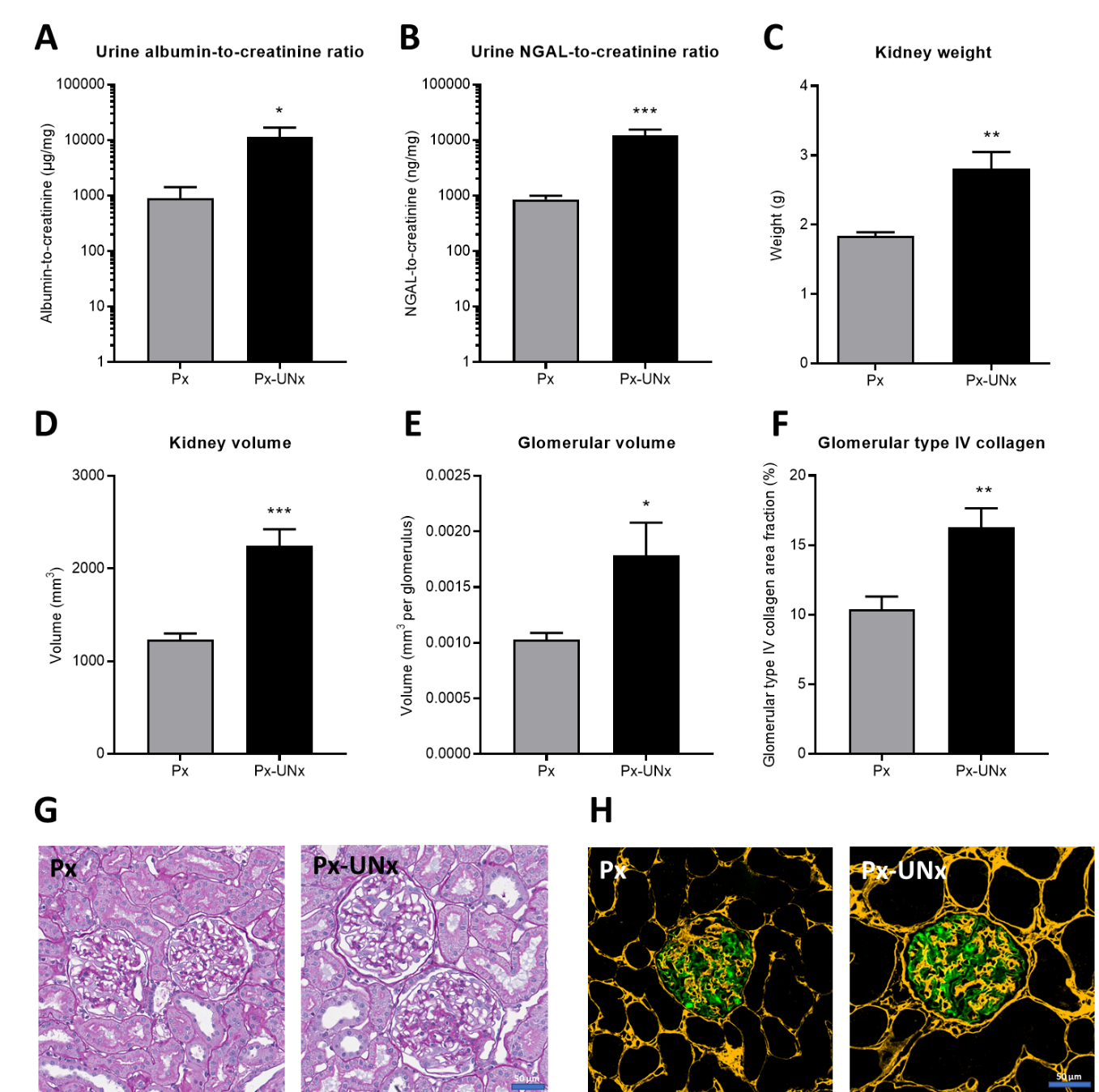
**

**Supplementary figure 2. Urine markers and renal histopathology from a pilot study of Px-UNx.** At 7-8 weeks of age, 17 rats underwent pancreatectomy (Px) alone or Px in combination with unilateral nephrectomy (UNx). Urine and renal samples were collected 11 weeks later to assess effects of unilateral nephrectomy (UNx) in pancreatectomized (Px) rats. Urine albumin-to-creatinine ratio (A) and urine NGAL-to-creatinine ratio (B) in spot urine samples at termination. Right kidney weight (C) at termination. Total kidney volume (D) and individual glomerular volume (E) of the right kidney as determined by stereology. Intra-glomerular collagen 4 (F) in the right kidney as determined by morphometric analysis. Representative images of Periodic Acid-Schiff (PAS) stained kidney sections (G) and kidney sections stained for co-localization type IV collagen (yellow) and podocin (green, H). Data are mean + SEM (n=7-10). Unpaired t-test; *p<0.05, **p<0.01, ***p<0.001 vs. Px.
